# Germline variants in genes of the subcortical maternal complex and Multilocus Imprinting Disturbance are associated with miscarriage/infertility or Beckwith–Wiedemann progeny

**DOI:** 10.1186/s13148-022-01262-2

**Published:** 2022-03-22

**Authors:** Pierpaola Tannorella, Luciano Calzari, Cecilia Daolio, Ester Mainini, Alessandro Vimercati, Davide Gentilini, Fiorenza Soli, Annalisa Pedrolli, Maria Teresa Bonati, Lidia Larizza, Silvia Russo

**Affiliations:** 1 Research Laboratory of Medical Cytogenetics and Molecular Genetics, IRCCS Istituto Auxologico Italiano, Milan, Italy; 2 Pediatric Unit, ASST Mantova, Borgo Mantovano, Italy; 3 Bioinformatics and Statistical Genomics Unit, Istituto Auxologico Italiano IRCCS, Milan, Italy; 4 Department of Brain and Behavioral Sciences, University of Pavia, Pavia, Italy; 5 Medical Genetic Unit, S. Chiara Hospital APSS, Trento, Italy; 6 Division of Pediatric, S. Chiara Hospital APSS, Trento, Italy; 7 Clinic of Medical Genetics, IRCCS Istituto Auxologico Italiano, Milan, Italy

**Keywords:** Multiple loci imprinting disorders, Maternal effect genes, Subcortical maternal complex genes, Beckwith–Wiedemann syndrome, Multiple miscarriages, Infertility

## Abstract

Beckwith–Wiedemann syndrome (BWS, OMIM # 130650) is an imprinting disorder, associated with overgrowth and increased risk of embryonal tumors. Patients carrying hypomethylation in the *KCNQ1OT1*:TSS DMR (11p15.5) show MLID (Multilocus Imprinting Disturbance) upon epimutations at other imprinted regions. Few cases of BWS MLID’s mothers with biallelic pathogenetic variants in maternal effect genes, mainly components of the subcortical maternal complex, are reported. We describe two families, one with a history of conception difficulties with a novel homozygous nonsense *NLRP2* variant and another experiencing 8 miscarriages with a compound heterozygous *PADI6* variant.

**Supplementary Information:**

The online version contains supplementary material available at 10.1186/s13148-022-01262-2.

## Introduction

Beckwith–Wiedemann Syndrome (BWS; OMIM#130650) is an overgrowth disorder characterized by heterogeneous clinical expression and complex molecular etiology. Distinctive clinical features are macroglossia, abdominal wall defects, lateralized overgrowth, enlarged abdominal organs, and increased risk of embryonal tumors during early childhood. The molecular defects consist in (epi)genetic deregulation of the 11p15.5 region containing two clusters of imprinted genes that play a key role in the regulation of fetal and postnatal growth: *H19/IGF2:IG-DMR* (IC1), methylated on the paternal allele, and *KCNQ1OT1:TSS-DMR* (IC2), methylated on the maternal allele which regulate the expression of *IGF2* and *CDKN1C* genes, respectively. More than 50% of BWS patients display hypomethylation of IC2 (Loss of Methylation, LoM) on the maternal allele, and 5% show hypermethylation in the paternal IC1 (Gain of Methylation, GoM) [1].

IC2-LoM in BWS patients may be associated with epimutations in additional differentially methylated regions (DMRs) involved in other imprinting disorders, giving rise to a phenomenon defined as Multilocus Imprinting Disturbance (MLID). Several reports on MLID frequency in IC2-LoM BWS cases picture a variable but significant frequency, from 30 to 50%. However, knowledge on the etiology and the clinical impact of MLID is still under study [1].

Genomic sequencing of MLID patients’ mothers highlighted the occurrence of pathogenic variants in genes transcribed by the maternal genome and deposited in the oocyte, where they persist until the first phases of embryogenesis [2]. When the zygote genome is not yet transcriptionally active, these maternal genes contribute to the zygote genome activation (ZGA) and passage to early embryo, hence being named Maternal-effect Genes (MEGs). Out of MEGs, the subcortical maternal complex (SCMC), a large protein complex expressed in oocytes and preimplantation embryos and functionally conserved across mammals, turned out to be associated with MLID in humans [2]. The SCMC, located in the cortex of the oocyte cytoplasm and enduring in the embryo up to the blastocyst stage, plays a role in processes as meiotic spindle formation and positioning, regulation of translation, organelle redistribution, and epigenetic reprogramming. Even not all the SCMC players have been identified, at present the proteins proved to be part of the complex are *NLRP2, NLRP4f, NLRP5,* and *NLRP9b*, all belonging to the NLRP (nucleotide-binding oligomerization domain, leucine-rich repeat, and pyrin domain-containing protein) family, *PADI6* (peptidyl arginine Deiminase 6), *KHDC3L* (KH domain containing 3), *OOEP* (oocyte expressed protein), *TLE6*, (transducin-like enhancer of Split 6), and *ZBED3* (zinc finger, *BED* type containing 3). In animals first and recently in humans, it has been demonstrated that the destruction of the complex causes sterility/sub-sterility in females, often conceiving embryos unable to go beyond the first cell division [3].

Loss-of-function mutations in maternal trans-acting SCMC genes in healthy women with disturbed imprinting in their offspring or women with infertility or recurrent miscarriages rather than hydatidiform moles would point to a causative role of SCMC variants in the MLID onset [2].

To date, pathogenic variants in maternal-effect genes in BWS patients with an IC2-LoM and MLID genotype have been disclosed in a limited number of studies. The reported cases include both biallelic and monoallelic variants, though the pathogenicity of the latter remains uncertain. The first description of SCMC variants in IC2-LoM BWS was a homozygous *NLRP2* frameshift in the mother of 2 BWS children, each inheriting a different *NLRP2* allele: consistent with a trans-acting mechanism of the germline variant, analysis of methylation at additional loci identified loss of methylation in *MEST:alt-TSS-DMR* [4]. Another family has been then reported with the same homozygous *NLRP2* variant, two BWS children, and a miscarriage history [5]. In both families, parents were consanguineous and Pakistani. A compound heterozygous *NLRP7* genotype was shared by two sisters experiencing recurrent miscarriages before the 7th gestational week, one of whom conceiving a BWS IC2-LoM MLID fetus, voluntarily terminated at the 19th week [5]. Furthermore, an *NLRP5* compound heterozygous genotype was reported in mothers of a BWS-MLID child in four independent families [6, 7] summing up to seven families documenting mothers with biallelic variants in *NLRP* genes and adverse reproductive outcomes and BWS MLID offspring.

Additionally, four families with BWS-MLID children and mothers, carriers of compound heterozygous *PADI6* variants [5, 8, 9] have been described. These data show a spectrum of conditions caused by SCMC alterations that impact oocyte developmental events leading to infertility and miscarriages or biallelic hydatidiform moles, as well as live births though with Beckwith–Wiedemann and MLID.

## Materials and methods

Out of a cohort of 195 BWS children with a molecular diagnosis of *KCNQ1T1*:TSS-DMR locus hypomethylation, a subset of patients distinguished for maternal reproductive issues was investigated for the occurrence of MLID phenomenon. Out of these, 10 mothers underwent exome sequencing. Two cases are here described due to the impressive reproductive history of their mothers.

Probands: MLID was investigated by methylation array Bead Chip array 450 K (Illumina) in case 1, while case 2 underwent MS-MLPA (MCR-Holland, ME034) and MS-SNuPE targeted approach. Details on methods are reported in Additional file 1.

Probands’ mothers: whole-exome was interrogated on DNAs, and the analysis was performed prioritizing genes coding for components of the SCMC or related genes, Maternal effect genes (MEGs), genes involved in oocyte maturation and oocyte-embryo transition, associated with reproductive issues, with (potential) roles in the control of genomic imprinting and involved in DNA methylation (Additional file 2).

Pathogenic variants were validated by Sanger sequencing (Additional file 3).

## Results

**Fig. 1:**
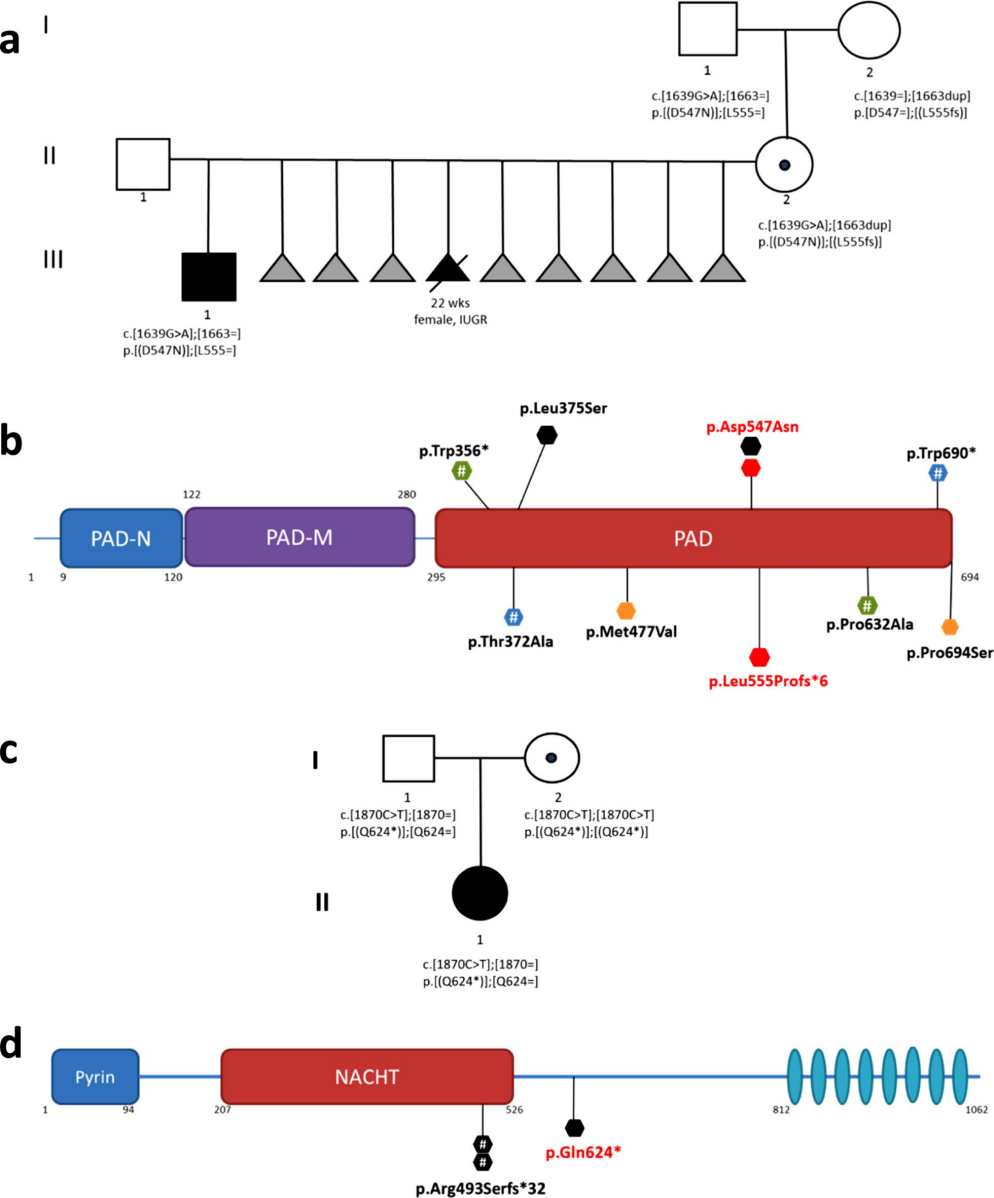
Pedigrees and schematic of PADI6 and NLRP2 proteins. **a** three generation pedigree of family 1 showing a BWS-MLID child and multiple miscarriages; **b** scheme of PADI6 protein: variants associated with BWS-MLID are indicated by hexagons. Same color hexagons point to compound heterozygous women. All the *PADI6* variants in BWS-MLID families cluster in the PAD domain of the protein. **c** Pedigree of family 2; **d** Diagrammatic structure of the human NLRP2 protein showing Pyrin, NACHT, and leucine-rich repeat domains. Reported variants are associated with BWS-MLID progeny. Grey triangles indicate miscarriages with unknown phenotypes. The dot within the circle indicates a woman carrier of SCMC variants. Genotype is reported for each studied individual; variants described in this study are in red characters. Each hexagon represents a family. #Variants associated with recurrence of BWS children in the family

Table 1 shows the DMRs with an altered methylation profile identified in the two probands. MLID epigenetic changes in BWS1 hit 10 additional imprinted loci, none of which is recognized as “disease” locus, while in BWS2 four loci associated with IDs, including *GNAS* and *PLAGL1*, are deregulated. *KCNQ1T1*:*TSS-DMR* locus hypomethylation was excluded in the probands’ mothers. Families’ pedigrees are shown in Fig. 1a, c.

**Table 1:** Summary of the hypomethylated and hypermethylated DMRs in the BWS1 and BWS2 probands

	*KCNQ1OT1*:TSS-DMR	*PLAGL1*:alt-TSS-DMR	*PEG3*:TSS-DMR	*GNAS-NESP*:TSS-DMR	*GNAS* locus	*PPIEL*:Ex1-DMR	*DIRAS3* locus	*ERLIN2:*Int6-DMR	*ZDBF2/GPR1*:IG-DMR	*WDR27*:Int13-DMR	*FANCC*:Int1-DMR	*INPP5F*:Int2-DMR	*ZNF597*:TSS-DMR	*ZNF331* locus	*WRB*:alt-TSS-DMR	*SNU13*:alt-TSS-DMR
Parental origin	M	M	M	P	M	M	M	M	P	M	M	M	P	M	M	M
BWS 1	↓	–	–	–	–	↓	↓	–	↑	↓	↓	↓	↑	↓	↓	↓
	− 40%					− 30%	− 28%		17%	− 41%	− 36%	− 22%	28%	− 30%	− 34%	− 29%
BWS 2	↓	↓	↓	↑	↓	n.t	–	↓	n.t	–	↓	n.t	n.t	n.t	–	–
	− 40%	− 40%	− 40%	25%	− 25%			− 15%			− 38%					

Each locus shows one DMR, apart (a) *GNAS*, including in the fourth column the paternal DMR and in the fifth one, the three maternal DMRs, and (b) *ZNF331* and *DIRAS3* including 2 DMRs**.** M: maternal origin of methylation, P: paternal origin of methylation, ↓: hypomethylation; ↑: hypermethylation: the percentage of hypo- or hyper-methylation at each locus compared to the relative median values of controls is indicated below the arrow, –: expected methylation pattern; nt: not tested. Cut-offs referred to each technique MS-MLPA, SNuPE and methylation array Bead Chip array 450 K are reported in Additional file 1

**Case 1** is a 13-year-old boy. At birth, (38 + 6 weeks), he displayed normal growth parameters (weight 2920 g (− 0.88 SDS), length 49 cm (− 0.46 SDS), and cranial circumference 35 cm (− 0.29 SDS)), macroglossia requiring frenotomy, umbilical hernia and a facial naevus simplex. Placenta analysis showed a mesenchymal dysplasia, which summed up to the previous newborn features addressed to a clinical diagnosis of Beckwith–Wiedemann syndrome. According to the consensus criteria [1], he achieved a score of 6. At age 2, lateralized overgrowth and a light leg length discrepancy became evident (< 1 cm), and worsened with the growth, leading to several hemiepiphysiodesis interventions. At this age, after the molecular diagnosis of *KCNQ1OT1:TSS-DMR* hypomethylation, a genome-wide methylation array analysis allowed to disclose 12 additional deregulated DMRs, in keeping with the MLID phenomenon (Table 1). At birth, his mother was 27 and presented hip dysmetria (about 1 cm). Since then, she had 8 miscarriages within the first trimester of pregnancy, and one abortion due to fetal malformations (IUGR, heart disease, and dysmorphisms) at the 22^a^ week of gestation (Fig. 1a). The couple then asked for genetic counseling to understand the cause of the recurrent fetal demise, but karyotype and CGH-array on the fetus did not reveal any chromosomal numeric or structural rearrangements. WES analysis on proband’s mother disclosed two potentially pathogenic variants in the *PADI6* gene. One variant, the missense NM_207421:c.1639G>A:p.Asp547Asn (rs150981529) is very rare (MAF = 0.00051, GnomAD), and its pathogenicity is not defined. Despite prediction tools (PolyPhen-2 = Benign, SIFT = tolerated, and CADD = 2.86) predicting it as potentially benign, the variant is classified as VUS according to the ACMG criteria (PM2, PM3, PP2, BP4), and it has been previously reported as probably pathogenic [5]. The second variant is a never reported frameshift variant in exon 15 (NM_207421.4:c.1663dupC:p.Leu555ProfsTer6; rs766500048). The missense variant is inherited from father, while the frameshift variant from mother (Fig. 1a, Additional file 3), proving the biallelic genotype of II-2. A schematic of PADI6 protein including all the pathogenic variants described in BWS families, and this study is displayed (Fig. 1b and Additional file 4).

**Case 2** is a 5-year-old girl born at 41 + 3 weeks of gestation with a birth weight of 3010 g (− 0.96 SDS), length of 49 cm (− 0.73 SDS), and cranial circumference of 35 cm (0.63 SDS). BWS features included neonatal hypoglycemia, macroglossia, and naevus flammeus consistent with a clinical score of 4 and a diagnosis of classic BWS. Multilocus analyses showed deregulations in 8 additional DMRs (Table 1). The proband's mother was 33 years old at the baby’s birth. The baby was naturally conceived, after 14 years of conception attempts, including one first-level and two second-level unsuccessful assisted fertilizations (Fig. 1c). Mother’s WES analysis revealed only a homozygous nonsense variant in the *NLRP2* gene (NM_017852.5:c.1870C>T:p.Gln624Ter). The variant leads to a stop codon in exon 5, and a predicted truncated protein, missing the C-terminal leucine-rich repeats (LRR). Never reported in the literature and databases, it is classified as pathogenic, according to the ACMG criteria, PVS1, PM2, PM3 [10]. Sanger sequencing showed the heterozygous variant both in the girl and her father (Fig. 1c, Additional file 3). Although the parents have denied possible consanguinity, we cannot exclude it with certainty. An inbred genotype for the girl’s mother was excluded as the ratio of homozygous/total variants is in the range of that of unrelated subjects.

## Discussion

Multilocus Imprinting Disorder is a signature of patients with one main ID, who have hypo or, rarely, hypermethylation in DMRs at other loci. The frequency of cases with MLID varies according to the syndrome, from the highest values of 50–70% in patients with Transient Neonatal Diabetes Mellitus (TNDM) to the rare cases in Angelman and Prader–Willi syndromes. In Beckwith–Wiedemann probands, MLID has been reported in 30–50% of patients with IC2-LoM [2]. The etiology of multiple epimutations featuring this phenomenon remains unclear. The finding of loss-of-function mutations in maternal trans-acting SCMC genes in healthy women with IDs and MLID offspring and infertility/recurrent miscarriages rather than hydatidiform moles would point to a causative role of SCMC variants in the MLID onset [2].

Aiming at contributing to the knowledge of this challenging epigenetic phenomenon, we explored the occurrence of pathogenic variants in SCMC genes in ten unrelated mothers of IC2-LoM and MLID-BWS children with classic phenotype. SCMC pathogenetic variants were disclosed only in two women, both experiencing an extremely troubled reproductive history. We disclosed a compound heterozygous *PADI6* genotype in one mother and a novel homozygous nonsense variant in the *NLRP2* gene in the other one. Both genes have been associated with infertility in mice. *PADI6* absence in knockout mice led to the dispersal of cytoskeletal sheets in oocytes, and in infertile *PADI6* compound-heterozygous women oocytes underwent a reduced amount of RNA polymerase II, essential for ZGA and consequent direct cleavages, which arrest embryo development [10]. Miscarriages of our patient’s mother may be considered as a milder expression of the same phenomenon.

Female mice *Nlrp2*^−/−^ showed atresia of ovarian follicles, reduced fertility and delayed or failed blastocyst formation, prenatal or perinatal death of the progeny with various growth and developmental defects. Lack of *Nlrp2* was found to causes methylation alterations in both maternal and paternal imprinted loci, caused by increased expression and mislocation of *Dnmt1* in the *Nlrp2*^−/−^ embryos [3]. Matching these findings, our mother was unable to conceive after 10 years and three failed ART attempts and the child shows several hypo/hyper-methylation alterations at maternal and paternal alleles. The overall number of cases with MEGs variants associated with BWS-MLID would thus include: **i)** 3 families with *NLRP2* homozygous variants, two with two affected children [4, 5] and our family segregating a novel variant to the only BWS child (Fig. 1d), **ii)** 4 mothers with compound heterozygous *PADI6* genotype [5, 8, 9] and our family 1. Interestingly, all the *PADI6* variants fall within the PAD domain and 2 out of 5 families generated 2 children with BWS. To note that 4/8 families with pathogenic variants in these two genes had 2 BWS children with MLID (Fig. 1). Clinical and molecular reports on new “familial” BWS cases has the family-centered aim of providing adequate genetic counseling.

Focusing on MLID, both BWS1 and BWS2 displayed a relevant number of deregulated DMRs, not affecting the same loci (Table 1) as to suggest a link of the entity of the deregulation to the presence of pathogenic variants.

To further our comprehension of the cascade of effects linking SCMC pathogenic variants to BWS-MLID, SCMC genes should be sequenced in mothers of IC2-LoM BWS children experiencing multiple miscarriages or sub-fertility.

## Supplementary Information

**Additional file 1.** Supplementary methods.

**Additional file 2.** Table S2. List of prioritized genes in WES analysis.

**Additional file 3.** Sanger sequencing and segregation of variants within each family.

**Additional file 4 ** Summary of genomic variants reported in *NLRP2* and *PADI6* genes.

## Data Availability

NGS data have been uploaded and are available at the public repository for research data Harvard Dataverse https://doi.org/10.7910/DVN/ATBYSZ. All other data are within the main text or its Additional Files.
