# Supplementary material for: Germline variants in genes of the subcortical maternal complex and Multilocus Imprinting Disturbance are associated with miscarriage/infertility or Beckwith–Wiedemann progeny
Source: Clin Epigenetics. 2022 Mar 22;14:43. doi: 10.1186/s13148-022-01262-2 (PMC8941822; doi:10.1186/s13148-022-01262-2)
Supplement: Supplementary file 1 — Additional file 1. Supplementary methods. [file 13148_2022_1262_MOESM1_ESM.docx]

**Clinical Epigenetics**

**Germline variants in genes of the Subcortical Maternal Complex and Multilocus Imprinting Disturbance are associated with Miscarriage/infertility or Beckwith-Wiedemann progeny**

Tannorella P, Calzari L, Daolio C, Mainini E, Vimercati A, Gentilini D, Bonati MT, Pedrolli A, Larizza L, Russo S.

**Supplementary Methods**

**DNA extraction**

DNA was extracted from peripheral blood lymphocytes of probands and their parents (Automated Extractor, Tecan Group Ltd. and Wizard Genomic DNA Purification Kit (Ref: A1125), Promega). Quality control and quantification of DNA were assessed by visualization of genomic DNA (gDNA) on 1% agarose gel electrophoresis and by using NanoPhotometer Pearl (Implen GmbH).

**Bisulfite conversion**

For each methylation assay, bisulfite conversion was carried on a maximum amount of 600 ng of gDNA treated by using the EZ DNA Methylation Kit (Ref: D5001, Zymo Research Corporation) according to the manufacturer’s protocol. Specific incubation conditions (Illumina Protocol) were always applied. To evaluate conversion efficiency and bsDNA integrity, a single-strand quantification of bisulfite converted DNA (bsDNA) was performed by using NanoPhotometer Pearl (Implen GmbH). Fragmented or too diluted DNA samples were discarded and then reprocessed.

**BeadChip 450K array: Stochastic Epigenetic Mutations (SEMs) analysis**

Genome-wide methylation analysis was performed by using the Infinium HumanMethylation 450K BeadChip (Illumina). The array-based procedure was carried out following the manufacturer’s instructions and using Illumina-supplied reagents and conditions. The control cohort consists of 44 methylation profiles of age-matched individuals: of these, 17 were healthy pediatric samples randomly distributed on microarrays together with cases to minimize batch effects.

Briefly, to identify DMRs with an aberrant methylation profile, a Stochastic Epigenetic Mutations (SEMs) analysis (Gentilini et al, 2015, Guida et al, 2021) was performed: the analysis was carried out at single sample level detecting, for each CpG site, the methylation values outside a “reference” methylation range obtained by the methylation profiles from the 44 controls’ cohort and calculated as follows: upper value=Q3+(k*IQR); lower value=Q1-(k*IQR); where Q1 is the first quartile, Q3 third quartile, IQR (Interquartile range)=Q3-Q1 and k=3. SEMs of single cases were classified as hyper-methylated or hypo-methylated compared to the relative probe median values of controls. Variability of methylation levels was also studied in the control populations for all the probes by applying SEM analysis and using each control as a case. A total of 689 CpG sites, distributed over 45 DMRs (genomic coordinates are provided below – Table 1), were investigated. Each DMR was considered as aberrantly methylated (deregulated) when more than 20% of CpG sites within the DMR showed extreme outlier methylation levels.

| Table 1  List of the known imprinted differentially methylated regions (45 DMRs) | | | | | | |  |
| --- | --- | --- | --- | --- | --- | --- | --- |
|  |  |  |  |  |  |  |  |
| Imprinted DMR | **Chr** | **# CpG** | **# 450K probes** | **Meth. origin** | **Germline derived** | **Associated disease** | **Genomic coordinates** |
| *PPIEL*:Ex1 | 1 | 39 | **4** | **M** | **Oocyte gDMR** | MLID | 40024626-40025540 |
| DIRAS3:TSS | 1 | 88 | **16** | **M** | **Oocyte gDMR** | MLID | 68515433-68517545 |
| DIRAS3:Ex2 | 1 | 39 | **8** | **M** | **Oocyte gDMR** | MLID | 68512505-68513486 |
| GPR1-AS:TSS | 2 | 86 | **3** | **M** | **Oocyte gDMR** |  | 207066967-207069445 |
| ZDBF2/GPR1:IG | 2 | 439 | **6** | **P** | **Sperm gDMR**-sec. DMR |  | 207114583-207136544 |
| NAP1L5:TSS | 4 | 57 | **13** | **M** | **Oocyte gDMR** | MLID | 89618184-89619237 |
| VTRNA2-1:DMR | 5 | 76 | **17** | **M** | **Oocyte gDMR** |  | 135414802-135416645 |
| FAM50B:TSS | 6 | 90 | **24** | **M** | **Oocyte gDMR** | MLID | 3849082-3850359 |
| PLAGL1:alt-TSS | 6 | 143 | **15** | **M** | **Oocyte gDMR** | **TNDM; MLID** | 144328078-144329888 |
| WDR27:Int13 | 6 | 58 | **2** | **M** | **Oocyte gDMR** | MLID | 170054504-170055618 |
| GRB10:alt-TSS | 7 | 171 | **9** | **M** | **Oocyte gDMR** | MLID | 50848726-50851312 |
| PEG10:TSS | 7 | 119 | **53** | **M** | **Oocyte gDMR** | MLID | 94285537-94287960 |
| MEST:alt-TSS | 7 | 226 | **54** | **M** | **Oocyte gDMR** | MLID | 130130122-130134388 |
| HTR5A:TSS | 7 | 55 | **6** | **M** | **Oocyte gDMR** |  | 154862719-154863382 |
| ERLIN2:Int6 | 8 | 37 | **7** | **M** | **Oocyte gDMR** | MLID | 37604992-37606088 |
| PEG13:TSS | 8 | 193 | **8** | **M** | **Oocyte gDMR** | MLID | 141108147-141111081 |
| FANCC:Int1 | 9 | 26 | **2** | **M** | **Oocyte gDMR** | MLID | 98075400-98075744 |
| INPP5F:Int2 | 10 | 52 | **3** | **M** | **Oocyte gDMR** |  | 121578046-121578727 |
| *H19/IGF2*:IG | 11 | 250 | **47** | **P** | **Sperm gDMR** | **SRS/BWS** | 2018812-2024740 |
| *IGF2*:Ex9 | 11 | 63 | **10** | **P** | No-sec. DMR | **SRS/BWS** | 2153991-2155112 |
| *KCNQ1OT1*:TSS | 11 | 192 | **28** | **M** | **Oocyte gDMR** | **BWS (rare Sotos)** | 2719948-2722259 |
| RB1:Int2 | 13 | 195 | **11** | **M** | **Oocyte gDMR** |  | 48892341-48895763 |
| MEG3:TSS | 14 | 188 | **33** | **P** | No-sec. DMR | **TS14/KOS14** | 101290524-101293978 |
| MKRN3:TSS | 15 | 109 | **12** | **M** | **Oocyte gDMR**-sec. DMR |  | 23807086-23812495 |
| MAGEL2:TSS | 15 | 51 | **6** | **M** | No-sec. DMR |  | 23892425-23894029 |
| NDN:TSS | 15 | 108 | **8** | **M** | No-sec. DMR |  | 23931451-23932759 |
| SNRPN:alt-TSS | 15 | 19 | **8** | **M** | No-sec. DMR |  | 25068564-25069481 |
| SNRPN:Int11 | 15 | 44 | **4** | **M** |  |  | 25093008-25193829 |
| SNRPN:Int12 | 15 | 45 | **4** | **M** |  |  | 25123027-25123905 |
| SNURF:TSS | 15 | 113 | **6** | **M** | **Oocyte gDMR** | **PWS/AS** | 25200004-25201976 |
| IGF1R:Int2 | 15 | 55 | **7** | **M** | **Oocyte gDMR** |  | 99408496-99409650 |
| ZNF597:3' DMR | 16 | 29 | **2** | **M** | **Oocyte gDMR** |  | 3481801-3482388 |
| ZNF597:TSS | 16 | 76 | **12** | **P** | No-sec. DMR |  | 3492828-3494463 |
| ZNF331:alt-TSS1 | 19 | 125 | **8** | **M** | **Oocyte gDMR** | MLID | 54040510-54042212 |
| ZNF331:alt-TSS2 | 19 | 102 | **4** | **M** | **Oocyte gDMR** |  | 54057086-54058425 |
| PEG3:TSS | 19 | 221 | **35** | **M** | **Oocyte gDMR** | MLID | 57348493-57353271 |
| MCTS2P:TSS | 20 | 47 | **9** | **M** | **Oocyte gDMR** | MLID | 30134663-30135933 |
| NNAT:TSS | 20 | 135 | **36** | **M** | **Oocyte gDMR** |  | 36148604-36150528 |
| L3MBTL1:alt-TSS | 20 | 84 | **25** | **M** | **Oocyte gDMR** | MLID | 42142365-42144040 |
| GNAS-NESP:TSS | 20 | 257 | **22** | **P** | No-sec. DMR | **PHP** | 57414039-57418612 |
| GNAS-AS1:TSS | 20 | 128 | **60** | **M** | **Oocyte gDMR** | **PHP** | 57425649-57428033 |
| GNAS-XL:Ex1 | 20 | 200 | **6** | **M** | **Oocyte gDMR** | **PHP** | 57428905-57431463 |
| GNAS A/B:TSS | 20 | 198 | **39** | **M** | No-sec. DMR | **PHP** | 57463265-57465201 |
| WRB:alt-TSS | 21 | 43 | **4** | **M** | **Oocyte gDMR** | MLID | 40757510-40758276 |
| SNU13:alt-TSS | 22 | 63 | **8** | **M** | **Oocyte gDMR** | MLID | 42077774-42078873 |

The methylation thresholds of DMRs reported in Table 1 of the main Text were calculated from the control population (n=44). The mean of the upper and lower methylation values and the median (Q2) (plus relative standard deviations) of the CpG sites, are reported below:

- PPIEL:Ex1: Upper-value:0.93(sd:0.05), Median:0.67(sd:0.09), Lower-value:0.43(sd:0.17);
- DIRAS3-locus: Upper-value:0.75(sd:0.13), Median:0.53(sd:0.09), Lower-value:0.33(sd:0.08);
- ZDBF2/GPR1:IG: Upper-value:0.78(sd:0.13), Median:0.69(sd:0.06), Lower-value:0.34(sd:0.13);
- WDR27:Int13: Upper-value:0.9(sd:0.05), Median:0.6(sd:0.09), Lower-value:0.5(sd:0.13);
- ERLIN2:Int6: Upper-value:0.88(sd:0.06), Median:0.55(sd:0.11), Lower-value:0.31(sd:0.1);
- FANCC:Int1: Upper-value:0.738sd:0.03), Median:0.5(sd:0.01), Lower-value:0.26(sd:0.03);
- INPP5F:Int2: Upper-value:0.89(sd:0.17), Median:0.66(sd:0.05), Lower-value:0.45(sd:0.08);
- ZNF597:TSS: Upper-value:0.65(sd:0.1), Median:0.43(sd:0.09), Lower-value:0.23(sd:0.11);
- ZNF331-locus: Upper-value:0.69(sd:0.14), Median:0.44(sd:0.13), Lower-value:0.21(sd:0.23);
- WRB:alt-TSS: Upper-value:0.73(sd:0.14), Median:0.52(sd:0.10), Lower-value:0.33(sd:0.11);
- SNU13:alt-TSS: Upper-value:0.75(sd:0.18), Median:0.48(sd:0.08), Lower-value:0.21(sd:0.29).

**Methylation-specific multiplex ligation-dependent probe amplification (MS-MLPA) analyses.**

MRC_Holland ME030-BWS/RSS (lot C3-1212 and later) was applied for the first molecular diagnoses. The ME034 B1-0219 kit was used for a first level of MLID assessment. For each experiment batch, at least three normal age- and tissue-matched control samples were processed in parallel, according to the manufacturer’s instructions. Raw data were analyzed by using Coffalyser.Net Software (Version 140701 - MRC-Holland) which provides both information about copy number variations (normal ratio range: 0.85-1.15) and methylation values. Healthy individuals' methylation profiles (tissue-matched) (n=70) were used to establish specific cut-off values for abnormal methylation profiles at each locus. These MS-MLPA thresholds were set as 3 standards deviations from the mean (calculated from controls):

- KCNQ1OT1:TSS: Upper-value:0.66, Lower-value:0.42, Mean:0.54(sd:0.04);
- PLAGL1:alt-TSS: Upper-value:0.57, Lower-value:0.41, Mean:0.49(sd:0.026);
- PEG3:TSS: Upper-value:0.63, Lower-value:0.41, Mean:0.52(sd:0.036);
- GNAS-NESP:TSS: Upper-value:0.63, Lower-value:0.45, Mean:0.54(sd:0.03);
- GNAS-AS1:TSS: Upper-value:0.64, Lower-value:0.46, Mean:0.55(sd:0.03);
- GNAS-XL:Ex1. Upper-value:0.61, Lower-value:0.41, Mean:0.51(sd:0.035);
- GNAS A/B:TSS: Upper-value:0.60, Lower-value:0.40, Mean:0.50(sd:0.035).

**Methylation-sensitive single-nucleotide primer extension (MS-SNuPE) analyses**

The MS-SNuPE assay was carried out as described in (Begemann M et al, 2012)) with some modifications. Seven loci were subjected to analysis: *KCNQ1OT1*:TSS-DMR, *DIRAS3*:Ex2, *WDR27*:Int13, *ERLIN2*:Int6, *FANCC*:Int1,SNU13:alt-TSS, and *WRB*:alt-TSS. A single representative CpG was chosen for each locus. The choice of the position of the primers was made taking into account the absence of common SNPs (MAF>0.001) (dbSNP 1.4.7). Genomic coordinates of PCR and target CpG sites are provided below (Table 2). After bisulfite conversion, 50-100ng of bsDNA were amplified by using QIAGEN Multiplex PCR Kit (QIAGEN) to a final volume of 50 ul according to the manufacturer’s instructions. PCR multiplex program is available upon request. Two different batches of multiplex amplification were created by pooling the relative primers. Two independent rounds of amplification were then performed: to remove unincorporated primers and dNTPs, 2 ul of illustra ExoProStar (GE Healthcare) were added to 5 ul of multiplex PCR products and incubated at 37°C for 15 min and 80°C for 15 min for enzymes inactivation. The sequencing reaction was carried out by using ABI PRISM SNaPshot Multiplex Kit (Applied Biosystems). 3 ul of purified PCR products were processed according to manufacturer’s instructions (defaults SNaPshot program). 1 unit of Illustra Shrimp Alkaline Phosphatase (GE Healthcare) was added to the mix and incubated at 37°C for 60 min and at 80°C for 15 min to inactivate the enzyme. 2.5 ul were denatured and run on an AB-3500 Genetic Analyzer (Applied Biosystems), together with 0.1 ul of GeneScan™ 120 LIZ™ dye (Applied Biosystems) as size standard to a final volume of 10 ul with Hi-Di™ Formamide (Applied Biosystems). A methylation index (MI) was determined by annotating peak areas of methylated and unmethylated alleles through GeneMapper software (Applied Biosystems). MI= peak area of methylated (blue) allele/(peak area of methylated (blue) allele + peak area of unmethylated (green) allele). For each DMR, a reference methylation range (MI ± 3SD) was set as a normal range by considering and analyzing 31 healthy controls (range available upon request). At least 4 controls were randomly processed together with samples in each experiment. Mean values of the control MI were set to 0.5. The scaled MI values of patients falling outside 3 standard deviations from the mean were taken into account for altered methylation patterns.

The MS-SNuPE thresholds are reported below:

- DIRAS3 locus: Upper-value:0.68, Lower-value:0.32, Mean:0.50(sd:0.06);
- ERLIN2:Int6: Upper-value:0.65, Lower-value:0.35, Mean:0.50(sd:0.05);
- WDR27:Int13: Upper-value:0.77, Lower-value:0.23, Mean:0.50(sd:0.09);
- FANCC:Int1: Upper-value:0.74, Lower-value:0.26, Mean:0.50(sd:0.08);
- WRB:alt-TSS: Upper-value:0.68, Lower-value:0.32, Mean:0.50(sd:0.06);
- SNU13:alt-TSS: Upper-value:0.89, Lower-value:0.11, Mean:0.50(sd:0.13).

| **Table 2 – Genomic coordinates of PCR amplicons and CpG target sites of the MS-SNuPE technique** | | | | | | |
| --- | --- | --- | --- | --- | --- | --- |
| **SnuPE Set 1** | | | | | | |
|  | **Amplification** | | | | | **SNaPshot Sequencing**  **CpG site** |
|  | **Locus** | **Chromosome** | **Start** | **End** | **bp** | **CpG Coordinate (hg19)** |
|  | DIRAS3:Ex2 | chr1 | 68512618 | 68512893 | 275 | chr1:68512807-68512808 |
|  | ERLIN2:Int6 | chr8 | 37605889 | 37606209 | 320 | chr8:37605978-37605979 |
|  | WDR27:Int13 | chr6 | 170055237 | 170055379 | 142 | chr6:170055332-170055333 |
|  | WRB:alt-TSS | chr21 | 40757479 | 40757749 | 270 | chr21:40757695-40757696 |
|  | KCNQ1OT1:TSS | chr11 | 2721176 | 2721297 | 121 | chr11:2721237-2721238 |
| **SnuPE Set 2** | | | | | | |
|  | **Amplification** | | | | | **SNaPshot Sequencing**  **CpG site** |
|  | **Locus** | **Chromosome** | **Start** | **End** | **bp** | **CpG Coordinate (hg19)** |
|  | FANCC:Int1 | chr9 | 98075351 | 98075616 | 265 | chr9:98075492-98075493 |
|  | SNU13:alt-TSS | chr22 | 42078639 | 42078791 | 153 | chr22:42078752-42078753 |

We inform that all reported cut-offs refer specifically to our experimental batch and therefore, using different experimental conditions (different control cohorts, protocols, reagents, and instruments), they could accordingly slightly vary.

***WES (Whole-Exome Sequencing) analysis***

Whole-exome was interrogated on genomic DNAs at BIODIVERSA srl Service (Milan, Italy), using the SureSelect Human All Exon V7 library and the Illumina HiSeq X platform. Bioinformatic analyses were carried out at Bioinformatic Unit of the Istituto Auxologico Italiano (Milano, Italy). Fastq data were aligned to the reference genome assembly GRCh37/hg19 ) using the Maximal Exact Matches algorithm in the Burrows-Wheeler Aligner (BWA) (v.0.7.10). PCR duplicates removal was performed by using Picard (v1.119) (picard.sourceforge.net/). Unified Genotyper (GATK v3.7) was used to locally realign Insertion/Deletions (InDels) and recalibrate base quality scores. Variants were visually inspected using the Integrative Genomics Viewer (IGV, Broad Institute), and further annotated using wANNOVAR (<http://wannovar.wglab.org/>). For quality filtering, variants with coverage of >20x and variant allele frequency more than or equal to 0.35 were selected. Next, we designed a panel of genes (**Supplementary File 2**), including: genes coding for components of the SCMC or related genes; Maternal effect genes (MEGs); genes involved in oocyte maturation and oocyte-embryo transition; genes associated with reproductive issues; genes with (potential) roles in the control of genomic imprinting; genes involved in DNA methylation.

Variants were also filtered according to minor allele frequency (MAF) > 1% in the 1000 Genomes, Genome Aggregation Database (gnomAD), and Exome Aggregation Consortium (ExAC) databases.

To evaluate the potential impact of missense variant pathogenicity, we combined the Poly-Phen-2, SIFT and CADD algorithms; while for the intronic and synonym variants in silico splicing prediction was carried out using, NNSplice predictor (Reese et al, 1997).

Finally, the interpretation of the variants was based on the classification by the Varsome database (Kopanos et al, 2019) and according to the ACMG–AMP (American College of Medical Genetics and Genomics/Association for Molecular Pathology) guidelines (Richards et al, 2015) as pathogenic, likely pathogenic, with uncertain significance, and likely benign or benign. Benign and Likely benign variants were excluded. Pathogenic variants were validated by Sanger sequencing (**Supplementary File 3**).

**References**

Gentilini D, Garagnani P, Pisoni S, Bacalini MG, Calzari L, Mari D, et al. Stochastic epigenetic mutations (DNA methylation) increase exponentially in human aging and correlate with X chromosome inactivation skewing in females. Aging (Albany NY). 2015;7(8):568-78.

Guida V, Calzari L, Fadda MT, Piceci-Sparascio F, Digilio MC, Bernardini L, et al. Genome-Wide DNA Methylation Analysis of a Cohort of 41 Patients Affected by Oculo-Auriculo-Vertebral Spectrum (OAVS). International journal of molecular sciences. 2021;22(3).

Begemann M, Leisten I, Soellner L, Zerres K, Eggermann T, Spengler S. Use of multilocus methylation-specific single nucleotide primer extension (MS-SNuPE) technology in diagnostic testing for human imprinted loci. Epigenetics. 2012;7(5):473-81.

Reese, MG, Eeckman, FH, Kulp, D, Haussler D. Improved splice site detection in Genie. Journal of Computational Biology 1997;4(3), 311-323.

Kopanos C, Tsiolkas V, Kouris A, Chapple CE, Albarca Aguilera M, Meyer R, et al. VarSome: the human genomic variant search engine. Bioinformatics. 2019;35(11):1978-80.

Richards S, Aziz N, Bale S, Bick D, Das S, Gastier-Foster J, et al. Standards and guidelines for the interpretation of sequence variants: a joint consensus recommendation of the American College of Medical Genetics and Genomics and the Association for Molecular Pathology. Genetics in medicine : official journal of the American College of Medical Genetics. 2015;17(5):405-24.
