# Supplementary material for: Germline variants in genes of the subcortical maternal complex and Multilocus Imprinting Disturbance are associated with miscarriage/infertility or Beckwith–Wiedemann progeny
Source: Clin Epigenetics. 2022 Mar 22;14:43. doi: 10.1186/s13148-022-01262-2 (PMC8941822; doi:10.1186/s13148-022-01262-2)

Family Case 1

PADI6 Variants: NM_207421: c.1639G>A:p.Asp547Asn (rs150981529)
c.1663dupC:p.Leu555ProfsTer6 (rs766500048)

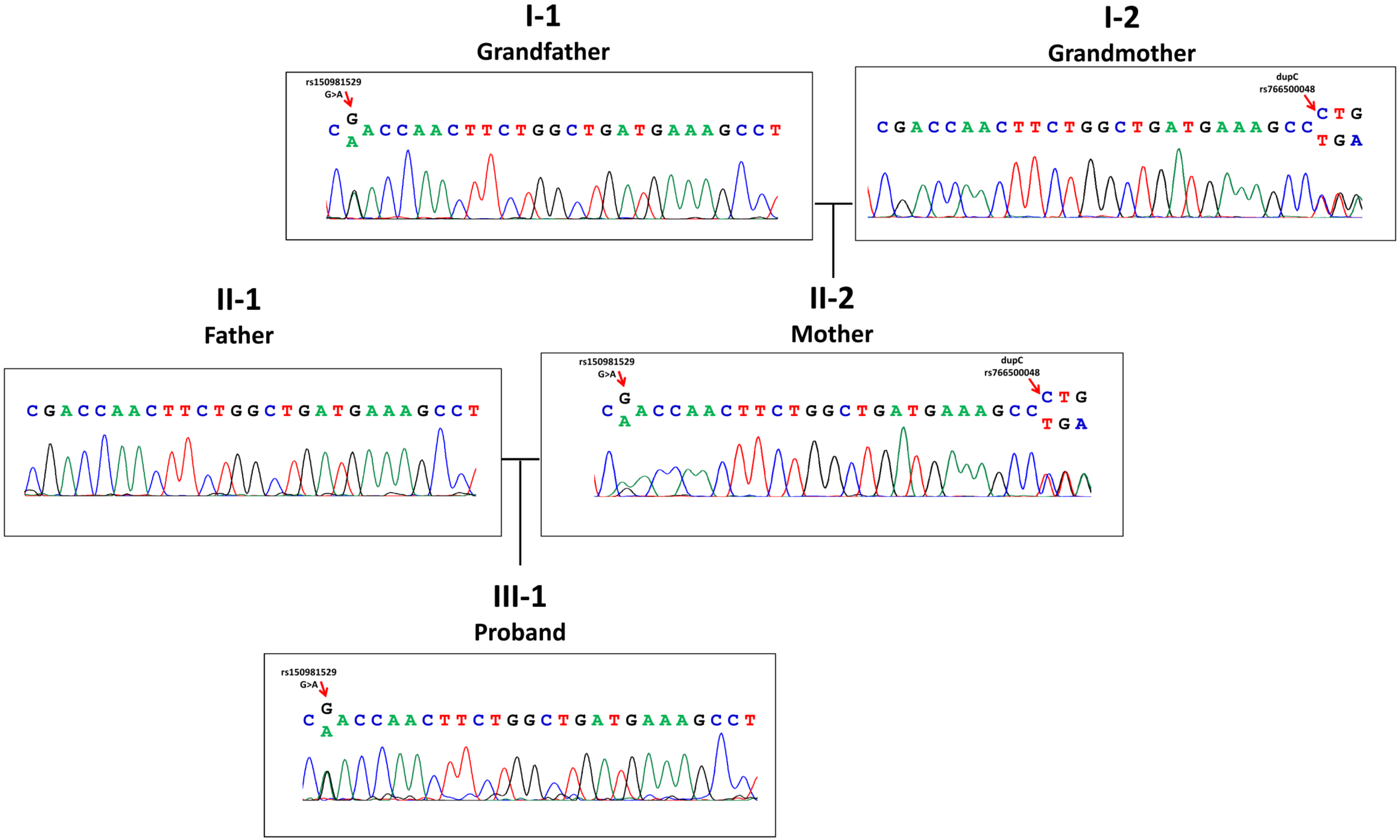

Family Case 2

NLRP2 (NM_017852.5:c.1870C>T:p.Gln624Ter)

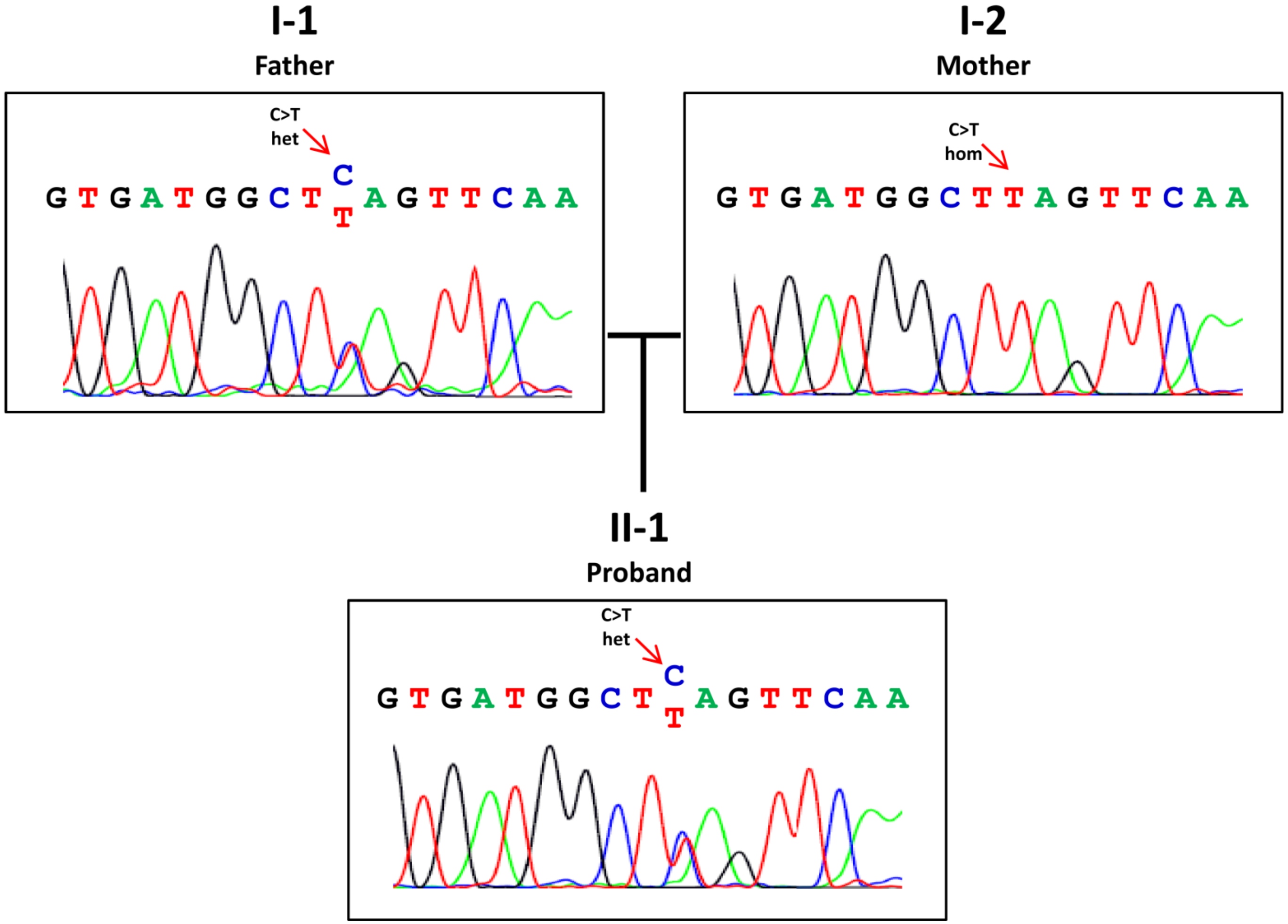

Supplement: Supplementary file 3 — Additional file 3. Sanger sequencing and segregation of variants within each family. [file 13148_2022_1262_MOESM3_ESM.pdf]
