# Supplementary material for: Germline variants in genes of the subcortical maternal complex and Multilocus Imprinting Disturbance are associated with miscarriage/infertility or Beckwith–Wiedemann progeny
Source: Clin Epigenetics. 2022 Mar 22;14:43. doi: 10.1186/s13148-022-01262-2 (PMC8941822; doi:10.1186/s13148-022-01262-2)
Supplement: Supplementary file 4 — Additional file 4 Summary of genomic variants reported in NLRP2 and PADI6 genes. [file 13148_2022_1262_MOESM4_ESM.docx]

| **Gene** | **Zygosity in mother** | **cDNA position** | **Protein position** | **rs** | **Allele frequency (gnomAD)** | **ACMG classification** | **Recurrence** | **abortions/ miscarriages** | **Reference** |
| --- | --- | --- | --- | --- | --- | --- | --- | --- | --- |
| *NLRP2* | homozygous | c.1479_1480del | p.Arg493Serfs*32 | rs758760659 | 0.0000756 | Pathogenic (PVS1, PM2, PM3) | 2 BWSp children | 3 | Begemann et al, 2018 |
|  | homozygous | c.1479_1480del | p.Arg493Serfs*32 | rs758760659 | 0.0000756 | Pathogenic (PVS1, PM2, PM3) | 2 BWSp children |  | Maher et al, 2009 |
|  | homozygous | c.1870C>T | p.Gln624Ter |  | none | Pathogenic (PVS1, PM2, PM3) |  |  | -- |
| *PADI6* | compound heterozygous | c.1114A>G  c.2069G>A | p.(Thr372Ala)  p.(Trp690*) |  | none  none | VUS (PM2, PM3, BP4)  Pathogenic (PVS1, PM2) | 2 BWSp children | 3 | Eggermann et al, 2021 |
|  | compound heterozygous | c.1124T>C  c.1639G>A | p.(Asp547Asn)  p.(Leu375Ser) | rs150981529  rs1470278066 | 0.000518  0.00000401 | VUS (PM2, BP4)  VUS (PM2, BP4) |  |  | Begemann et al, 2018 |
|  | compound heterozygous | c.1067G>A  c.1894C>G | p.(Trp356*)  p.(Pro632Ala) | -  rs755260464 | none  0.00000401 | Pathogenic (PVS1, PM2)  VUS (PM2, PM3, PP3) | 2 BWSp children |  | Cubellis et al, 2020 |
|  | compound heterozygous | c.1429A>G  c.2080C>T | p.(Pro694Ser)  p.(Met477Val) | rs1368496637  rs761556429 | 0.00000805  0.00000401 | VUS (PM2, BP4)  VUS (PM2, BP4) |  | 1 | Cubellis et al, 2020 |
|  | compound heterozygous | c.1639G>A  c.166dup | p.(Asp547Asn)  p.(Leu555Profs*6) | rs150981529  rs766500048 | 0.000518  0.00000803 | VUS (PM2, PM3, PP2, BP4)  Pathogenic (PVS1, PM2) |  | 9 | -- |
